# Facile synthesis of diverse graphene nanomeshes based on simultaneous regulation of pore size and surface structure

**DOI:** 10.1038/srep32310

**Published:** 2016-08-26

**Authors:** Jia Zhang, Huaibing Song, Dawen Zeng, Hao Wang, Ziyu Qin, Keng Xu, Aimin Pang, Changsheng Xie

**Affiliations:** 1 State Key Laboratory of Material Processing and Die & Mould Technology, Nanomaterials and Smart Sensors Research Laboratory, Department of Materials Science and Engineering, Huazhong University of Science and Technology, Wuhan 430074, PR China; 2 Hubei Collaborative Innovation Center for Advanced Organic Chemical Materials, Wuhan 430062, PR China; 3 Wuhan National Laboratory for Optoelectronics (WNLO), Huazhong University of Science and Technology, Wuhan 430074, PR China; 4 Hubei Institute of Aerospace Chemotechnology, No. 58, Qinghe Road, Xiangyang 441003, PR China

## Abstract

Recently, graphene nanomesh (GNM) has attracted great attentions due to its unique porous structure, abundant active sites, finite band gap and possesses potential applications in the fields of electronics, gas sensor/storage, catalysis, *etc*. Therefore, diverse GNMs with different physical and chemical properties are required urgently to meet different applications. Herein we demonstrate a facile synthetic method based on the famous Fenton reaction to prepare GNM, by using economically fabricated graphene oxide (GO) as a starting material. By precisely controlling the reaction time, simultaneous regulation of pore size from 2.9 to 11.1 nm and surface structure can be realized. Ultimately, diverse GNMs with tunable band gap and work function can be obtained. Specially, the band gap decreases from 4.5–2.3 eV for GO, which is an insulator, to 3.9–1.24 eV for GNM-5 h, which approaches to a semiconductor. The dual nature of electrophilic addition and oxidizability of HO^•^ is responsible for this controllable synthesis. This efficient, low-cost, inherently scalable synthetic method is suitable for provide diverse and optional GNMs, and may be generalized to a universal technique.

Graphene, the two-dimensional sp^2^-hybridized carbon with unique long-range π-conjugation, has been extensively studied in numerous intriguing applications due to its extraordinary thermal, mechanical, and electrical properties^1^,^2^,^3^,^4^. However, the zero band gap nature and chemical inertness of graphene have greatly hindered its applications in the fields requiring semiconducting properties and/or abundant active sites. Hence, a major focus of theoretical and experimental studies has been concentrated on band gap engineering^5^,^6^,^7^,^8^ and surface modification^9^ of graphene. Graphene nanomesh (GNM), a porous graphene network with plentiful in-plane nanopores on the conjugated carbon surface, is a “rising star” for graphene band gap engineering, first creatively reported by Duan and co-workers in 2010 10. Besides the adjustable band gap, GNM offers fascinating integrated features of large area, high specific surface area, unique porous structure, abundant superficial and border active sites, thus exhibiting promising prospects for widely applications including electronics, energy storage/conversion, gas sensor/storage/filtration, catalysis, *etc*.^11^,^12^,^13^,^14^,^15^. So far, most GNMs are fabricated by either template- or etching-method. Template-method is generally used in the preparation of large area GNMs with precisely controlled pore size and shape through adjusting the size of various templates^16^,^17^,^18^,^19^,^20^,^21^. Due to their homogeneity, the resultant GNMs are especially suitable for the electrical applications as a semiconductor with a constant band gap. Etching-method is a promising approach for large-scale and low-cost preparation for GNMs, and suitable for the applications requiring large quantity, high porosity and abundant active sites^22^,^23^,^24^,^25^,^26^,^27^,^28^,^29^,^30^. Noticeably, except for regulating pore size - essentially regulating neck width between neighbouring pores, the surface modification of functionalised graphene is another common method to modulate band gap, since the physicochemical properties of carbon materials are strongly dependent on the ratio of sp^2^ to sp^3^ bonds^31^, which embodied in the positive correlation between the optical gap of reduced graphene oxide (rGO) and the atomic ratio of O/C^32^,^33^, and the different roles of each oxygen functional groups in the optical gap regulation of rGO^34^. Moreover, the oxygen functional groups can also provide further chemical reaction/modification sites, good hydrophilia and consequent processibility, which are highly important for particle application and the materials’ extended research, like as a scaffold^35^,^36^. Although the previously reported synthetic methods of GNM have created an impressive success in the regulation of band gap by adjusting pore size/density, the conscious adjustment of surface structure, especially oxygen functional groups, of GNM is rarely concerned. Consequently, it is highly desirable to develop an effective method for mass preparation of versatile GNMs combining variable pore size and surface structure, thus providing diverse and optional band gap and surface chemical activity to match different applications.

The Fenton reaction, established in 1894 37, has been proven to be an effective method to degrade aromatic compound in wastewater by producing a variety of highly reactive hydroxyl radical (HO^•^)^38^,^39^,^40^. To some extent, graphene and its derivatives can also be considered as super-aromatic molecules, and have potential to react with the Fenton reagent. Recently, the Fenton reaction has been successfully employed to investigate the effect of hydroxyl radical (HO^•^) on the surface structure of multi-walled carbon nanotubes (MWNTs) and fabricate graphene quantum dots (GQDs)^41^,^42^. The relative reaction mechanism was also studied through both theoretical and experimental approach^43^. Moreover, compared with the classical Fenton method, the ferrioxalate-mediated photo-Fenton method^44^ exhibits much higher etching efficiency due to more efficient and stable HO^•^ production benefiting from the highly efficient regeneration of ferrous ions. To be specific, the classical Fenton method produces hydroxyl radical (HO^•^) mainly *via* the Haber-Weiss reaction, Eq. (1). Meanwhile, Fe(II) can be regenerated from Fe(III) through the Fenton-like reaction, Eq. (2), which can in turn react with hydrogen peroxide to sustainedly generate hydroxyl radicals. However, the reaction 2 is a wavelength dependent reaction (just below 313 nm), its rate constant k is several orders of magnitude slower than reaction 1, thus reaction 2 is the rate-limiting step^45^. Fortunately, the ferrioxalates are photochemically active and have nearly constant quantum yield (1.0–1.2 at 254–442 nm) for ferrous ion production in UV-Vis region according to Eqs (3–4). Therefore, the efficient etching reaction can be realized on account of the sufficient and continuously regenerated ferrous ion.
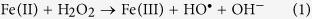

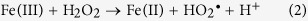

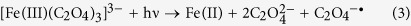

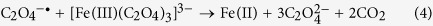


In this work, inspired by the effective reaction between carbonaceous materials and Fenton reagent in the previous works, we present a facile fabrication of diverse GNMs with simultaneously adjustable pore size and surface structure by using a ferrioxalate-mediated photo-Fenton method. This universal synthetic method overcomes the obstacles of zero band gap and chemical inertness of graphene simultaneously, and exploring their potentials for wide application.

## Results and Discussion

**Figure 1:**
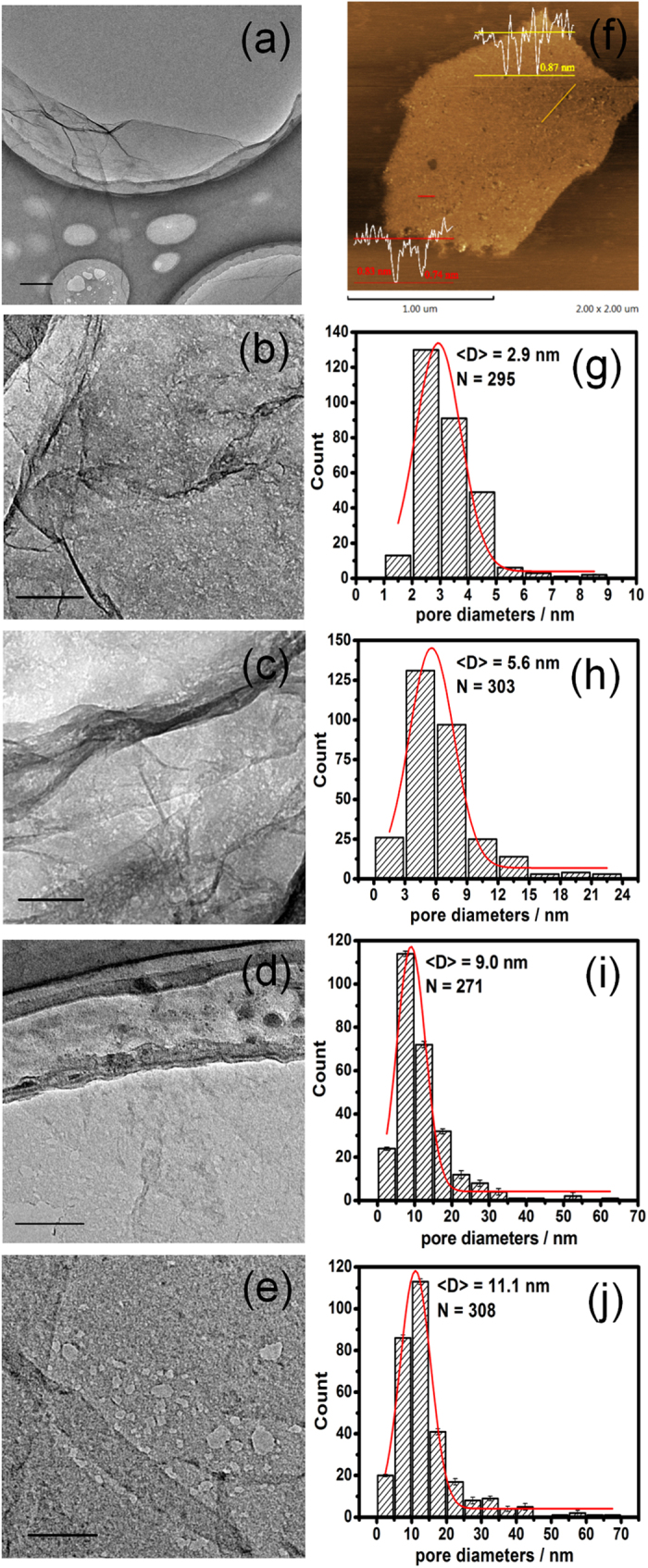
TEM images showed the evolution of GNM from GO (**a**) for different reaction times: 2 (**b**), 3 (**c**), 4 (**d**), and 5h (**e**). AFM image and height profiles (superimposed on the image) of GNM-4h (**f**). The statistical histograms combined with the corresponding Gaussian fitting of the nanopore sizes for GNMs-2, 3, 4, 5h, respectively (**g–j)**, <D> was the mean pore size, N was the sample number. Scale bars were 500 nm in (**a**) and 100 nm in (**b–e**).

Figure 1a–e display the TEM micrographs of morphological evolution of GNMs from GO. It was obvious that the pore size was enlarged gradually with the elongation of reaction time, eventually leading to biggish holes *via* the merger of adjacent pores. To determine the mean pore size of GNMs, three representative images of each sample were selected for statistical analysis, as shown in Fig. S1. Together with the Gaussian fitting, it could be seen that the mean pore size (<D>) of nanopore was increased from 2.9 to 11.1 nm with the reaction time increased from 2 to 5 h (Fig. 1g–j), indicating that the nanopore size in the GNM sheets can be regulated conveniently by timing the reaction. For the samples reacting more than 6 hours, severe tear would be found and the fragmentation of GO sheets would occur gradually (Fig. S2), which was beyond the scope of this article. The AFM image was also used to further confirm the formation of GNM (Fig. 1f), in which some nanopores were clearly seen on the sheet. Height profile along the lines showed successive fluctuations of approximate height of 0.74~0.87 nm, in agreement with the typical thickness of single-layer GO (~0.8 nm)^46^, further evidencing the successfully etching of GO.

**Figure 2:**
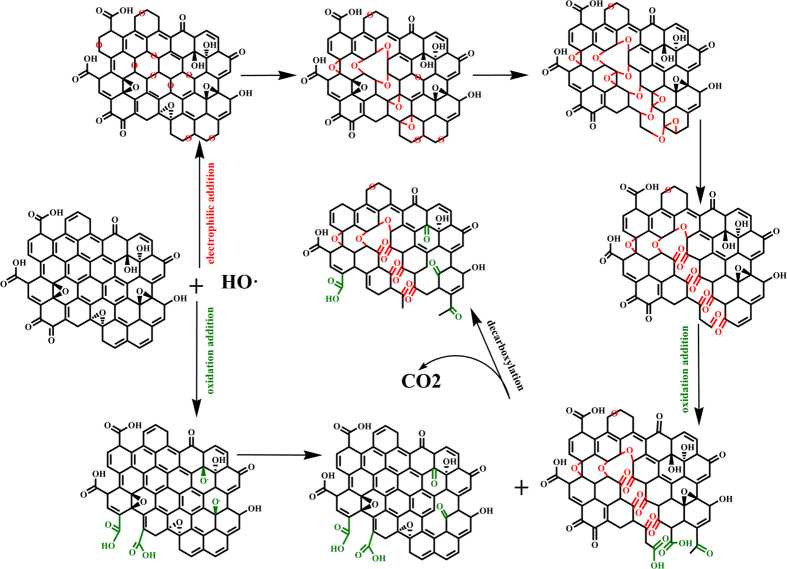
Schematic illustration of proposed mechanism describing the two competing reactions between GO and HO^•^ including electrophilic addition (red path) and oxidization reaction (green path).

Figure 2 illustrates the hypothesis of interactions between HO^•^ and GO. The structure of GO consisted of both sp^2^ graphitic domains and sp^3^ oxidized domains decorated with various oxygen functional groups, including hydroxyl, epoxides, carbonyl and carboxyl. It has been known that HO^•^ possess the dual nature of electrophilic addition and oxidizability, which play a vital role for punching holes on the basal plane of GO sheets. Firstly, the strong electron affinity (569.3 kJ) of HO^•^ enables it to preferentially attack high electron density sites, that is nonoxidized sp^2^ domains in the GO system, thus introducing large batch of epoxy groups, transforming partly planar sp^2^-hybridized carbon atoms to distorted sp^3^-hybridized carbon atoms, and eventually triggering the oxidative unzipping of GO^47^,^48^,^49^,^50^,^51^,^52^. As the simulated computation showed, oxygen atom were highly mobile on the graphitic platelet and prone to aggregation^33^, the clustering pattern as NNN EP trimer^51^ and linear configuration^47^,^48^,^53^ of epoxy groups on the GO could easily break the underlying C-C bonds at the aid of collectively lattice strain^51^, followed by a structural transformation into ether groups, and further into semiquinones, thereby introducing linear defects and cracks on the GNMs sheets with O-terminated edges. On the other hand, the aboriginal oxygen moieties in the sp^3^ domains of GO, together with that originated from above electrophilic addition reaction, were further transformed into higher oxidation states via oxidization reaction on account of strong oxidizability of HO^•^ with redox potential reach up to 2.8 eV, concretely converting hydroxyl to carbonyl, and further to carboxyl. Ultimately, a fraction of carboxyl groups was eliminated via decarboxylation with releasing of CO_2_. Obviously, these two reactions proceeded simultaneously and competed with each other, thus leading to the generation and enlargement of pores, as well as the variation of surface structure including the ratio of sp^2^/sp^3^ domains, the oxygen content, the species and content of various functional groups and defects. Therefore, it could be easily expected that the regulation of morphology and surface structure of GNMs can be realized by controlling reaction time and extent. The XPS, XRD and Raman were employed to monitor the evolutional process of surface structure of GNM.

**Figure 3:**
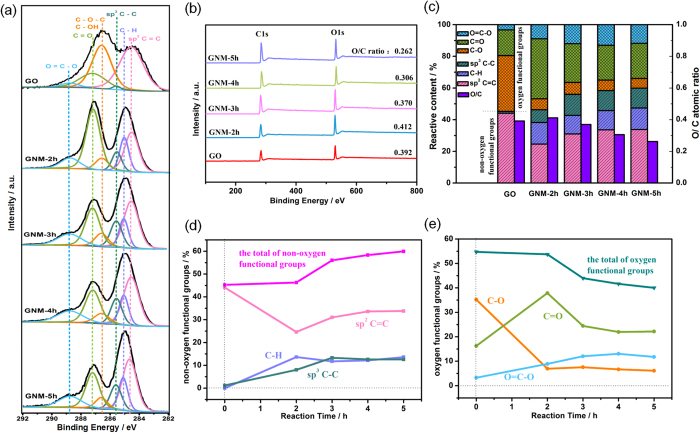
Deconvoluted C1s XPS spectra (**a**), XPS general spectra (**b**), the atomic ratio of O/C (violet) and the relative amounts of sp^2^ C=C, C-H, sp^3^ C-C, C-O/C-O-C, C=O and O=C-O bonds calculated based on the deconvoluted C1s XPS spectra (colours) illustrating the evolution of each group for GO and GNM-2, 3, 4, 5h (**c**), the relative amounts of non-oxygen functional groups (**d**) and oxygen functional groups (4) as a function of reaction time.

The XPS spectra is employed to unravel detailed information about the variation of chemical composition and structure of the GO and GNMs throughout the entire reaction process. The XPS general spectra of the GO and GNMs was presented in Fig. 3b. The deconvoluted C(1s) peaks of GO and GNMs showed peaks at 284.6, 285.1, 285.6, 286.6, 287.3 and 288.8 eV, corresponding to sp^2^ C=C, C-H defect, sp^3^ C-C, C-OH/C-O-C^24^, C=O and O=C-O, respectively, as shown in Fig. 3a. A variation of ±0.1 eV for the binding energies were accepted for the XPS deconvolution for all samples^54^. To quantitatively analyze the evolutional process of each bond along with the reaction process, the relative contribution of each bond to the C1s spectra of the GO and GNMs were calculated and presented in Fig. 3c, and the relative amount of the each and total of non-oxygen functional groups and oxygen functional groups as a function of the reaction time were presented in Fig. 3d,e, respectively. According to the above assumption, the electrophilic addition and the oxidative transformation of C=O to O=C-O would increase the O/C ratio of GNM, whereas the decarboxylic reaction would decrease the O/C ratio. Therefore, it was evident that electrophilic addition had a slight superiority in the early stage of the reaction (less than 2 hrs), and the decarboxylic reaction became dominate in the later stage of the reaction (more than 2 hrs). In the early stage, the large amount of sp^2^ C=C provided abundant attack sites for electrophilic addition, and resulting in dramatic decline of the relative amount of sp^2^ C=C of GNM-2h (Fig. c,d), as well as the slightly increased O/C ratio (Fig. b,c). However, most of the freshly generated C-O-C and aboriginal C-OH/C-O-C were concurrently transformed to C=O and even to O=C-O under the oxidative attack of HO^•^. As a result, a high proportion of C=O was seen in the GNM-2h, rather than C-O (Fig. 3c). In addition, a large percentage of C-H defects and sp^3^ C-C were introduced into GNM-2h, which normally resided at the edge sites and defect sites, corresponding to the appearance of numerous nanopores. In the later stage, the electrophilic addition reaction was weakened due to the decreased amount of vulnerable sp^2^ C=C bindings, combined with the faster reaction rate between HO^•^ and hydrophilic oxygen functional groups than hydrophobic sp^2^ domains^41^, consequently the active decarboxylic reaction led to the continuous declination of O/C ratio. The inapparent trends of the relative amounts of various oxygen functional groups were resulted from the complex and interlaced reactions of electrophilic addition, oxidation and decarboxylation. There seemed to be a dynamic equilibrium between three kinds of reactions. Additionally, it was worth mentioning that the increase of the relative amount of sp^2^ C=C after 2 hrs should be ascribed to the rapid elimination of oxygen functional groups, but not the restoration of sp^2^-C network.

**Figure 4:**
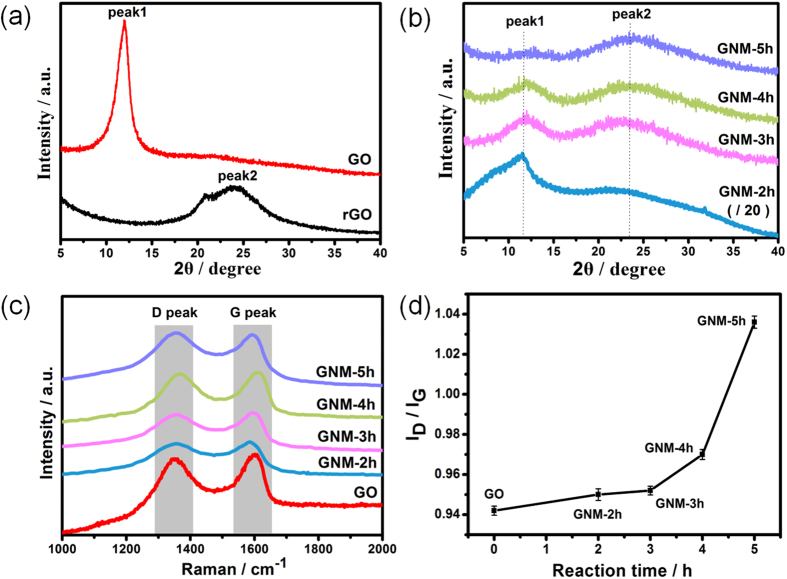
XRD patterns (**a**,**b**), Raman spectra (**c**), the intensity ratio of D/G peak (I_D_/I_G_) as a function of reaction time (**d**) of GO, GNM and rGO.

XRD analysis is a convenient method to investigate the structural change of carbon materials. The XRD patterns of GO, rGO and GNMs were demonstrated in Fig. 4a,b. The GO had a sharp peak near 11.8° (named peak1), indicating an interlayer distance of 7.5 Å, and the rGO had a broaden peak near 23.5° (named peak2), indicating an interlayer distance of 3.79 Å (Fig. 4a). It was known that this change was attributed to the removal of oxygen functional groups^32^. The Fig. 4b showed that all the XRD patterns of the GNMs possessed both of the two peaks simultaneously. Moreover, as reaction time was prolonged, the intensity of peak2 became stronger whereas the intensity of peak1 became weaker gradually, implying the stepwise elimination of oxygen functional groups, and resulting in the reduction process. Additionally, both peaks of all GNMs were broadening, implying that abundant defects and disorders were introduced into the GNMs system. All these results were consistent with the XPS results.

**Figure 5:**
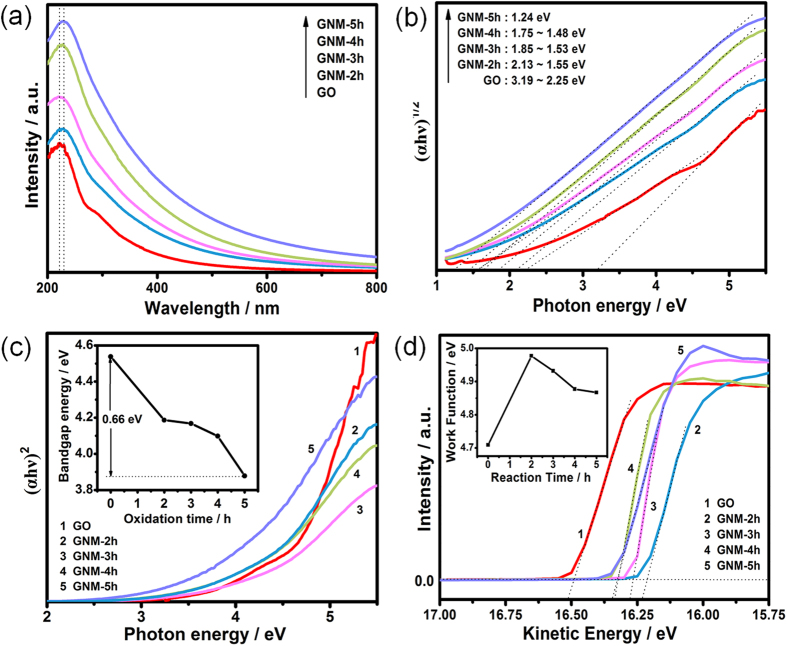
UV-Vis adsorption spectra (**a**), plots of (αhν)^1/2^ against the photon energy (hν) for indirect band gap deduction (**b**), plots of (αhν)^2^ against the photon energy (hν) for direct band gap deduction and the direct band gap as a function of reaction time (inset) (**c**), UPS spectra in the high kinetic energy cutoff region and the work function as a function of reaction time (inset) (d) of GO and GNMs.

Raman spectroscopy is an ideal characterization tool for studying the atomic structure of carbon materials. As shown in Fig. 4c,d, the two representative peaks of carbon materials, i.e., the G peak around 1580–1600 cm^−1^ and the D peak around 1350 cm^−1^, were observable in the spectrum of the GO and GNMs. The G peak is generally assigned to the first-order scattering of the E_2g_ phonons of sp^2^-hybridized carbon atoms, and the D peak is originated from the breathing mode of κ-point phonons of A_1g_ symmetry of the local defects and disorders involved in sp^3^-hybridized carbon bonds such as hydroxyl and/or epoxide bonds^55^. Therefore, the intensity ratio of D/G peak (I_D_/I_G_) is often considered as a measure of the disorder in the carbon material. In this work, the I_D_/I_G_ ratio increased continuously with the reaction time, from 0.942 for GO to 1.036 for GNM-5h (Fig. 5b), implying the gradually increase of defects and/or disorders. Combined with the XPS results, this can be attributed to both nanopores and increased sp^3^-hybridized geometry. Firstly, much more unsaturated carbon atoms were introduced on the rim sites of nanopores and tremendously increased disorders of the whole system. Meanwhile, lots of epoxide groups introduced through the electrophilic addition led to the fragmentation of nonoxidized sp^2^ domains by embedding weeny sp^3^ domains, introducing a large quantity of phase boundaries that were rich in defects. This can also be confirmed by Tuinstra and Koenig (TK)’s deduction (Equation 5)^56^, where La is the mean diameter of sp^2^ clusters and C (515.5 nm) ~44 Å, exhibiting the inverse relation of the I_D_/I_G_ ratio and the size of sp^2^ domains.
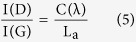


The band gap and work function are all important physical properties for graphene derivatives including GNM, especially for their applications in the fields of electronic, sensors, *etc*. Theoretical calculation has showed that the gap nature of GO would change from the direct gap to the indirect one with increasing oxygen content because of the change of the highest valence band state from C-C π orbital to the O_2p_ orbital^33^. Hence, for the results of UV-Vis spectra (Fig. 5a), we plotted the square root and square of the converted energy (αhν, where α is the absorption coefficient) against the photon energy (hν) to determine the indirect and direct gap transition energies of the GO and GNMs. The band gaps were deduced by extrapolating the linear region of the curves to the x-axis. Figure 5b displayed apparent indirect band gap energies of 3.19–2.25 eV for GO and 1.24 eV for GNM-5h. Figure 5c displayed apparent direct band gap energies of 4.538 eV for GO and 3.878 eV for GNM-5h. Both indirect and direct band gaps showed an evident decreasing tendency, approaching to the energy gap of semiconductor. The variation of band gap of the GNMs was the result of the combined action of the effective tuning of pore structure and surface oxygen functional groups. The effect of pore size was mainly attributed to quantum confinement effect^10^, and the effect of surface oxygen functional groups should be attributed to the localization effect, behaving in a similar way as doped semiconductors. The Fig. 5d depicted the UPS spectra of GO and GNMs in high kinetic energy regions. The secondary electron threshold energy was determined by extrapolating two dotted lines from the background and straight onset and reading the intersection value. The work function (Φ) was calculated as Φ = hν − E_th_, where hν and E_th_ were the photon energy of excitation light (He Ι discharge lamp, 21.2 eV) and the secondary electron threshold energy, respectively. The work function of GO was measured to be 4.71 eV. For GNMs, the work function was firstly increased to 4.98 eV for GNM-2h, and then decreased to 4.93, 4.88, 4.87 eV for GNM-3, 4, 5h, respectively, as shown in the inset of Fig. 5d. In conjunction with the XPS results, we inferred that the initial increase of work function of GNM-2h was attributed to the severe destruction of π-π conjugation and large increase of C=O and O=C-O content, which had stronger electron-withdrawing ability than C-O-C/C-OH, thus leading to the heavier p-type doping^57^. In comparison to GNM-2h, the slight decrease of work function of GNM-3, 4, 5h were mainly ascribed to the decrease of O/C ratio and comprehensive effect of the amount change of different types of oxygen functional groups^58^. Therefore, the band gap and work function of GNM can be progressively tuned by regulating the reaction time to meet different application demands.

## Conclusion

In conclusion, diverse GNMs with tunable pore size, surface structure, band gap and work function could be facilely fabricated from commercial GO *via* the ferrioxalate-mediated photo-Fenton method. The dual nature of electrophilic addition and oxidizability of HO^•^ was responsible for the generation and enlargement of nanopores, as well as the variation of surface structure including the ratio of sp^2^/sp^3^ domains, the oxygen content, the species and contents of various functional groups and defects, *etc*. Moreover, considering the high activity of HO^•^, this method also has potential to prepare similar porous materials, such as other carbonaceous materials, two-dimensional metal chalcogenide, *etc*.

## Methods

### Preparation of graphene nanomesh

Graphene oxide (GO) was produced from graphite power (325 mesh, Nanjing XFNANO Materials Tech Co. Ltd.) through a modified Hummer’s method with a peroxidation treatment^59^. GNMs were prepared through a ferrioxalate-mediated photo-Fenton method. Typically, 30 mg GO was added into 100 mL DI water to form a homogeneous suspension through ultrasonic process. Adjusting pH value of the solution with hydrochloric acid to 3.0–3.5, then 14 mg FeSO_4_∙7H_2_O, and 36 μL H_2_O_2_ were added into the mixture respectively, followed by ultrasonic treatment for one minute to trigger the reaction. Subsequently, 38 mg H_2_C_2_O_4_∙2H_2_O was added into the solution, and stirred for 15–20 min. The resulting suspension was radiated using a xenon lamp (CEL-HXUV300, UV Radiant Output: 6.6 W, UVREF: 200~400 nm) along with intense agitation, regular sampling at 2 h, 3 h, 4 h and 5 h. Finally, the products were centrifugally washed with DI water. The samples were collected and dried at 60 °C. The GNMs were designated as GNM-nh, where n refer to the time for UV radiation. rGO reduced by L-ascorbic acid was used for comparative analysis sometimes^60^.

### Characterization of graphene nanomesh

Transmission electron microscope (TEM) was employed to trace the morphology evolution of the GNMs with a JEM-2100 microscope working at 200 kV. The Atomic force microscopy (AFM) was utilized to confirm the detailed morphology using a SPM9700 (Shimadzu, Japan). X-ray diffraction (XRD) was obtained from GNMs powders using a Philips X′pert X-ray diffractometer with Cu Kα1 radiation (λ = 1.5406 Å) in the 2θ range from 5° to 40°. A Kratos XSAM800 spectrometer was employed for X-ray photoelectron spectroscopy (XPS) using Al Kα radiation (1486.6 eV) and Ultraviolet Photoelectron Spectroscopy (UPS) using He(Ι) (hν = 21.2 eV) discharge lamp as the energy source. Raman spectrum was carried out with a HORIBA Jobin Yvon Lab RAM HR operating at 532 nm wavelength at room temperature, scanning from 1000 cm^−1^ to 2000 cm^−1^. The peak intensities were employed directly to calculate the I_D_/I_G_ without the further processing of the initial data, such as integration and baseline subtraction. And ten parallel sampling data of each sample were collected to determine the error bars through the formula of standard deviation. The UV–Vis adsorption spectra was measured by using a PerkinElmer Lambda 35, scanned from 200 cm^−1^ to 1100 cm^−1^.

## Additional Information

**How to cite this article**: Zhang, J. *et al*. Facile synthesis of diverse graphene nanomeshes based on simultaneous regulation of pore size and surface structure. *Sci. Rep.* **6**, 32310; doi: 10.1038/srep32310 (2016).

## Supplementary Material

Supplementary Information
