# Supplementary material for: Facile synthesis of diverse graphene nanomeshes based on simultaneous regulation of pore size and surface structure
Source: Sci Rep. 2016 Aug 26;6:32310. doi: 10.1038/srep32310 (PMC4999802; doi:10.1038/srep32310)
Supplement: Supplementary Information [file srep32310-s1.pdf]

Electronic Supplementary Information

Facile synthesis of diverse graphene nanomeshes based on simultaneous regulation of pore size and surface structure

Jia Zhang,[1,2] Huaibing Song,[3] Dawen Zeng, [1,2*] Hao Wang,[1] Ziyu Qin,[1] Keng Xu,[1] Aimin Pang,[4] and Changsheng Xie[1]

[1)] State Key Laboratory of Material Processing and Die & Mould Technology, Nanomaterials and Smart Sensors Research Laboratory, Department of Materials Science and Engineering, Huazhong University of Science and Technology, Wuhan 430074, PR China.

[2)] Hubei Collaborative Innovation Center for Advanced Organic Chemical Materials, Wuhan 430062, PR China.

[3)] Wuhan National Laboratory for Optoelectronics (WNLO), Huazhong University of Science and Technology, Wuhan 430074, PR China.

[4)] Hubei Institite of Aerospace Chemotechnology, No. 58, Qinghe Road, Xiangyang 441003, PR China.

[*] Corresponding author: E-mail: dwzeng@mail.hust.edu.cn; Fax: +86-027-87543778; Tel: +86-027-87559835

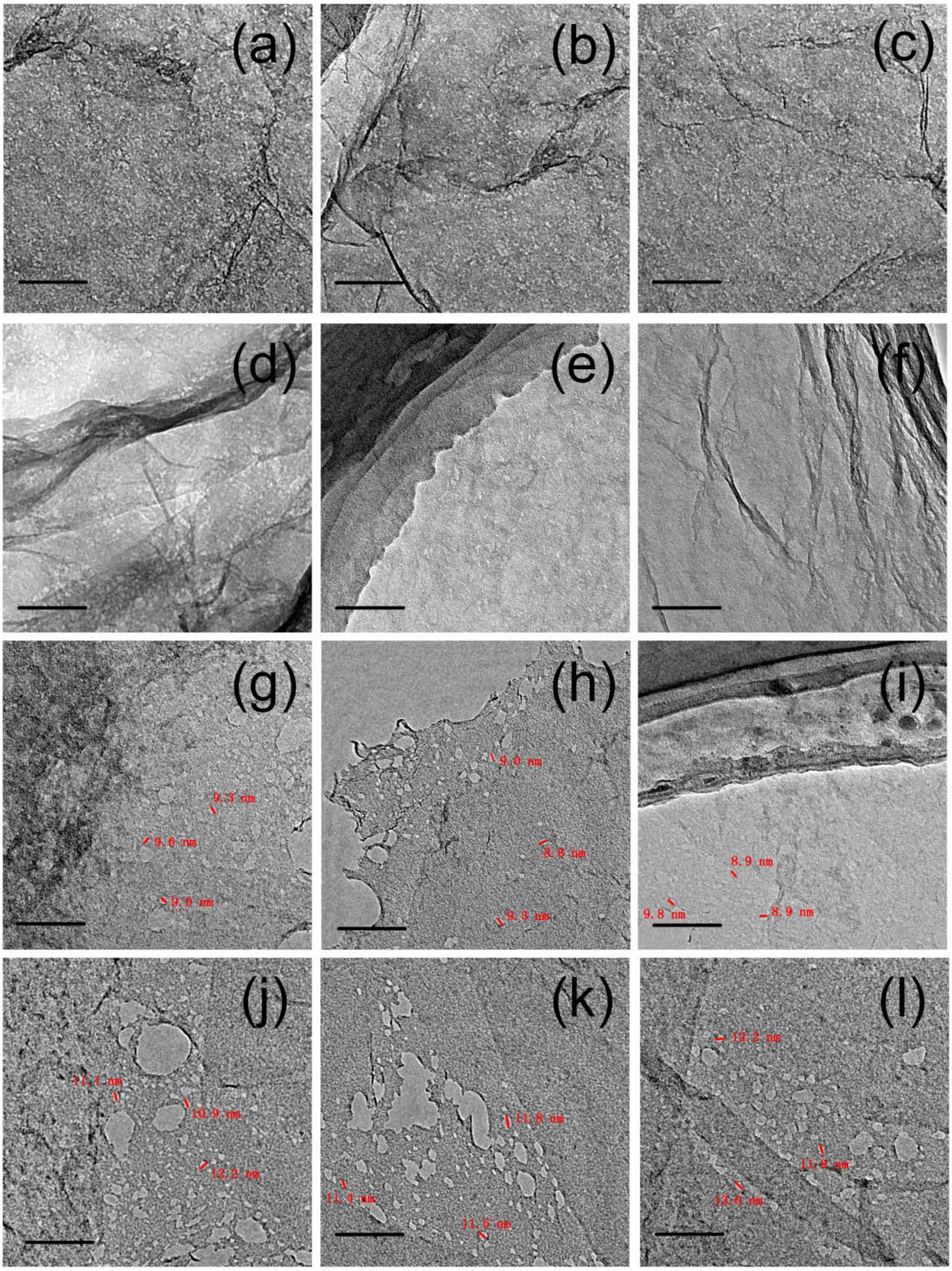

Fig S1. Three representative images of each sample were selected for statistical analysis, (a-c) for GNM-2h, (d-f) for GNM-3h, (g-i) for GNM-4h, (j-l) for GNM-5h. For GNM-4,5h, three nanopores of each image were marked with length dimension to facilitate the size estimate of adjacent nanopores. Scale bars are 100nm in (a-l).

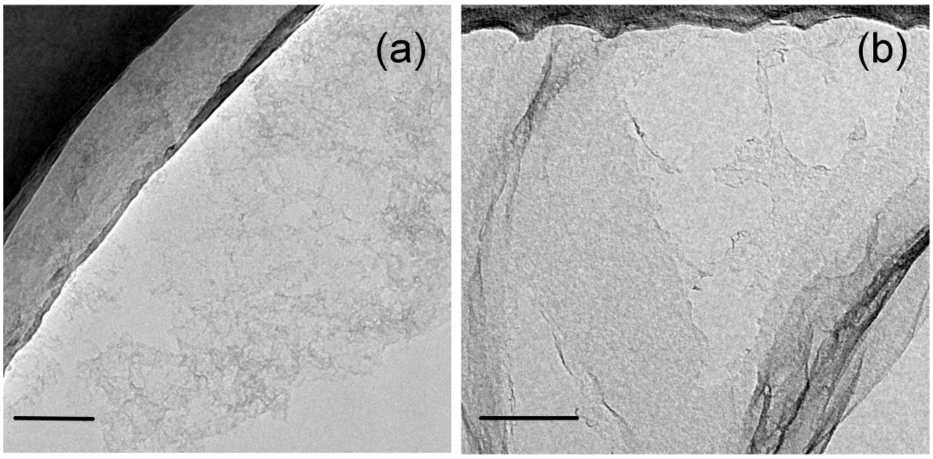

Fig S2. The TEM image for GNM-6h. Scale bars are 200nm in (a-b) and 100nm in (c).
